# Evaluating the effects of the novel GLP-1 analogue liraglutide in Alzheimer’s disease: study protocol for a randomised controlled trial (ELAD study)

**DOI:** 10.1186/s13063-019-3259-x

**Published:** 2019-04-03

**Authors:** Grazia Daniela Femminella, Eleni Frangou, Sharon B. Love, Gail Busza, Clive Holmes, Craig Ritchie, Robert Lawrence, Brady McFarlane, George Tadros, Basil H. Ridha, Carol Bannister, Zuzana Walker, Hilary Archer, Elizabeth Coulthard, Ben R. Underwood, Aparna Prasanna, Paul Koranteng, Salman Karim, Kehinde Junaid, Bernadette McGuinness, Ramin Nilforooshan, Ajay Macharouthu, Andrew Donaldson, Simon Thacker, Gregor Russell, Naghma Malik, Vandana Mate, Lucy Knight, Sajeev Kshemendran, John Harrison, David J. Brooks, Anthony Peter Passmore, Clive Ballard, Paul Edison

**Affiliations:** 1 Department of Medicine, Imperial College London, London, UK; 2 Centre for Statistics in Medicine, Nuffield Department of Orthopaedics, Rheumatology and Musculoskeletal Sciences, University of Oxford, Oxford, UK; 3 Southern Health NHS Foundation Trust, Havant, UK; 4 SW London and St George’s Mental Health Trust, London , UK; 5 Aston Medical school, Aston University, Birmingham, UK; 6 Brighton and Sussex University Hospitals NHS Trust, Brighton, UK; 7 South London and Maudsley NHS Foundation Trust, London, UK; 8 University College London and Essex Partnership University NHS Foundation Trust, Runwell, UK; 9 North Bristol NHS Trust, Bristol, UK; 10 Cambridgeshire and Peterborough NHS Foundation Trust, Peterborough, UK; 11 Black Country Partnership NHS Foundation Trust, West Bromwich, UK; 12 Northamptonshire Healthcare NHS Foundation Trust, Kettering, UK; 13 Lancashire Care NHS Foundation Trust, Preston, UK; 14 Nottinghamshire Healthcare NHS Foundation Trust, Nottingham, UK; 15 Centre for Public Health, Queen’s University Belfast, Belfast, UK; 16 Surrey and Borders Partnership NHS Foundation Trust, Leatherhead, UK; 17 NHS Ayrshire and Arran, Crosshouse, UK; 18 NHS Lanarkshire, Glasgow, UK; 19 Derbyshire Healthcare NHS Foundation Trust, Derby, UK; 20 Bradford District Care NHS Foundation Trust, Bradford, UK; 21 5 Boroughs Partnership NHS Foundation Trust, Warrington, UK; 22 Cornwall Partnership NHS Foundation Trust, Redruth, UK; 23 Somerset Partnership NHS Foundation Trust, Bridgwater, UK; 24 South Staffordshire and Shropshire Healthcare NHS Foundation Trust, Stafford, UK; 25 Alzheimer Center VUmc Amsterdam, Amsterdam, the Netherlands; 26 Institute of Psychiatry, Psychology & Neuroscience King’s College London, London, UK; 27 Newcastle University, Newcastle upon Tyne, UK; 28 School of Medicine, College of Biomedical and Life sciences, Cardiff University, Cardiff, CF14 4YS UK

**Keywords:** Alzheimer’s disease, Dementia, Randomised controlled trial, Liraglutide, Cerebral glucose metabolic rate

## Abstract

**Background:**

Liraglutide is a glucagon-like peptide-1 (GLP-1) analogue currently approved for type 2 diabetes and obesity. Preclinical evidence in transgenic models of Alzheimer’s disease suggests that liraglutide exerts neuroprotective effects by reducing amyloid oligomers, normalising synaptic plasticity and cerebral glucose uptake, and increasing the proliferation of neuronal progenitor cells. The primary objective of the study is to evaluate the change in cerebral glucose metabolic rate after 12 months of treatment with liraglutide in participants with Alzheimer’s disease compared to those who are receiving placebo.

**Methods/design:**

ELAD is a 12-month, multi-centre, randomised, double-blind, placebo-controlled, phase IIb trial of liraglutide in participants with mild Alzheimer’s dementia. A total of 206 participants will be randomised to receive either liraglutide or placebo as a daily injection for a year. The primary outcome will be the change in cerebral glucose metabolic rate in the cortical regions (hippocampus, medial temporal lobe, and posterior cingulate) from baseline to follow-up in the treatment group compared with the placebo group. The key secondary outcomes are the change from baseline to 12 months in *z* scores for clinical and cognitive measures (Alzheimer’s Disease Assessment Scale—Cognitive Subscale and Executive domain scores of the Neuropsychological Test Battery, Clinical Dementia Rating Sum of Boxes, and Alzheimer’s Disease Cooperative Study—Activities of Daily Living) and the incidence and severity of treatment-emergent adverse events or clinically important changes in safety assessments. Other secondary outcomes are 12-month change in magnetic resonance imaging volume, diffusion tensor imaging parameters, reduction in microglial activation in a subgroup of participants, reduction in tau formation and change in amyloid levels in a subgroup of participants measured by tau and amyloid imaging, and changes in composite scores using support machine vector analysis in the treatment group compared with the placebo group.

**Discussion:**

Alzheimer’s disease is a leading cause of morbidity worldwide. As available treatments are only symptomatic, the search for disease-modifying therapies is a priority. If the ELAD trial is successful, liraglutide and GLP-1 analogues will represent an important class of compounds to be further evaluated in clinical trials for Alzheimer’s treatment.

**Trial registration:**

ClinicalTrials.gov, NCT01843075. Registration 30 April 2013.

**Electronic supplementary material:**

The online version of this article (10.1186/s13063-019-3259-x) contains supplementary material, which is available to authorized users.

## Background

Alzheimer’s disease (AD) is a devastating progressive neurodegenerative disease and the most common form of dementia, affecting 10% of people over 65 years old and 40% of those over 85 years old. It is a major global healthcare burden. In 2015, an estimated 46.8 million people worldwide were living with dementia and the estimated global cost of dementia was US$818 billion [1]. The therapeutic options currently available only include symptomatic drugs that do not stop disease progression. There are no treatments available to slow disease progression or to prevent cognitive and functional deterioration. The identification of a safe and effective disease-modifying therapy is thus a key research priority [2].

There is compelling evidence that GLP-1 analogues exert influence on AD pathology by multiple mechanisms [3]. Given the urgent need for an effective treatment, we propose a phase IIb study to generate safety and efficacy data in people with AD for liraglutide. This compound is already licensed for treating type 2 diabetes mellitus and has shown promising results in a mouse model of AD and in a small group of AD participants [4]. If successful, this trial will highlight the importance of a definitive phase III trial to establish the efficacy of the compound in people with AD.

The rationale for using an antidiabetic drug in AD is based on the multiple pathophysiological connections that have been established between type 2 diabetes mellitus and AD. Type 2 diabetes has been identified as a risk factor for AD [5]. Insulin signalling is impaired in type 2 diabetes mellitus and is desensitised in AD brains [6]. Apart from controlling blood glucose, insulin has the general physiological profile of a growth factor. Neuronal insulin receptors can induce dendritic sprouting, neuronal stem cell activation, general cell growth, and repair [7–12]. Insulin and the related insulin-like growth factor (IGF-1) are both potent neuroprotective factors and regulate levels of phosphorylated tau [13, 14]. Insulin improves brain function such as attention, memory, and cognition in humans [15–18]. Nasal application of insulin, which allows it to enter the brain more directly than other application routes, has clear effects on attention and memory formation [17, 19, 20] but may be associated with risks, like inadvertent bouts of hypoglycaemia.

Moreover, a recent network meta-analysis has shown that, overall, antidiabetic agents significantly improved cognition in subjects with AD and mild cognitive impairment, indicating a pro-cognitive class effect of antidiabetic agents in these diseases [21].

However, it is conceivable that GLP-1 analogues can influence AD pathogenic mechanisms through pathways specifically related to the pharmacodynamics of the GLP-1 analogues, even in non-diabetic patients.

Among GLP-1 analogues, liraglutide is currently approved for treatment in type 2 diabetes and obesity in the EU and other countries. In diabetes, it has shown to be effective in glycaemic control, both as a monotherapy and in combination with other antidiabetic drugs, and to reduce cardiovascular risk [22, 23]. Its safety profile has been evaluated in different clinical trials and the most frequently reported adverse reactions during clinical trials were gastrointestinal disorders (nausea and diarrhoea) [24].

### Glucagon-like peptide-1 in the brain

Insulin is not the only glucostatic hormone that can act as a growth factor in the brain. Several parallel signalling systems also modulate blood glucose levels, such as the incretin hormone signalling pathways [25]. For example, glucagon-like peptide-1 (GLP-1) is an incretin hormone that binds to GLP-1 receptors mainly expressed on pancreatic beta-cells and the gastrointestinal system, which are G-protein coupled receptors [26].

GLP-1 receptors are also found in the brain [27–29]. Similar to insulin, GLP-1 in the brain is principally a growth factor that increases cell growth, proliferation, and repair and inhibits apoptosis [30]. It induces neurite outgrowth and protects against excitotoxic cell death and oxidative injury in cultured neuronal cells [31, 32]. In one study, neurons were protected against cell death induced by β-amyloid 1–42, and against oxidative stress and membrane lipid peroxidation caused by iron [27]. Mice that overexpress GLP-1 receptors in the hippocampus show increased neurite growth and improved spatial learning abilities [33].

Both the native peptide GLP-1 and long-lasting analogues such as extendin-4, Val(8)GLP-1, and liraglutide can cross the blood–brain barrier [26–29]. These analogues not only cross the blood–brain barrier after peripheral injection, but also show physiological effects in the brain by increasing neuronal progenitor proliferation, enhancing long-term potentiation in the hippocampus, improving learning, reducing plaque formation and inflammation in the brain, and even increasing neuroneogenesis [34].

GLP-1 analogues have neuroprotective effects in mouse models of AD. For example, one study gave chronic intraperitoneal injections of Val(8)GLP-1 for 3 weeks to a mouse model of AD that overexpressed the human Swedish mutated form of amyloid precursor protein (APP) and a human mutated form of presenelin-1 (PS1). The mice retained synaptic plasticity in their hippocampi, loss of which is an effect of plaque formation [35].

In another APP/PS1 mouse model of AD, liraglutide showed a range of protective effects when injected daily for 8 weeks at a dose comparable to that received by patients with diabetes. The key hallmarks of AD and neurodegeneration were reversed or improved, including β-amyloid synthesis, plaque formation, inflammation in the brain, synaptic loss, and memory impairment [36].

In different mouse models of diabetes, neuronal progenitor cell proliferation in the dentate gyrus was enhanced, and the number of young neurons in the dentate gyrus increased with systemic administration of both exenatide and liraglutide, indicating that additional repair processes had been activated in the brain [37, 38].

Insulin signalling was found to be desensitised in an ex vivo study of human brain tissue from AD patients. Liraglutide was able to reverse key biomarkers of insulin signalling desensitisation, such as the phosphorylation of insulin receptor β-chain IRβ pY^1162**/1163**^ and IRS-1 phosphorylation at S^616^ [39].

Together, these effects show a substantial reduction in key symptoms and hallmarks of AD in the presence of GLP-1 analogues. These analogues can cross the blood–brain barrier and do not affect blood glucose levels in normoglycaemic people [40]. As the GLP-1 analogue liraglutide has already been established on the market and shows few side effects, it is a promising AD treatment candidate.

A pilot clinical trial compared 18 AD participants treated with liraglutide with 20 AD participants treated with placebo. Six months of treatment with this GLP-1 analogue prevented decline of brain glucose metabolism, although no significant cognitive changes were observed, compared with the placebo group [4].

The primary objective of the ELAD trial is to evaluate the change in cerebral glucose metabolic rate after 12 months of treatment with liraglutide compared to the placebo. Secondary objectives include the evaluation of change in cognitive measures, MRI changes, microglial activation, amyloid and tau changes, and the incidence and severity of treatment-emergent adverse events.

## Methods and design

### Study design

ELAD is a 12-month, multi-centre, randomised, double-blind, placebo-controlled, phase IIb trial in participants with very mild AD dementia. Eligible participants will be randomised on a 1:1 ratio to receive liraglutide (1.8 mg) or matching placebo. A total of 206 participants will be recruited from sites across the UK, from local Memory Clinics and national databases, like “Join dementia research” (www.joindementiaresearch.nihr.ac.uk) All participants will start with a dose of 0.6 mg, and the dose will be escalated to 1.8 mg within 4 weeks. Participants who do not tolerate 1.8 mg will stay on 1.2 mg for an extra 2 weeks, and then two more attempts will be made to increase the dose to 1.8 mg. If 1.8 mg is still not tolerated, the participants will remain on 1.2 mg throughout the remainder of the trial.

### Outcome measures

Outcome measures in the ELAD trial include both biomarkers and clinical measure changes from baseline to follow up.

The primary outcome of the ELAD trial is as follows:

- The change in cerebral glucose metabolic rate in the cortical regions (hippocampal, medial temporal lobe, and posterior cingulate) from baseline to follow-up (12 months) in the treatment group, compared with the placebo group. This will be measured using [18F]fluorodeoxyglucose positron emission tomography (FDG-PET) at baseline and at 12 months.

Secondary outcomes are as follows:

- The change in cognitive and functional abilities from baseline to 12 months, measured as changes of *z* scores for the Alzheimer’s Disease Assessment Scale—Cognitive Subscale and Executive domain scores of the Neuropsychological Test Battery (ADAS Exec), Clinical Dementia Rating Sum of Boxes (CDR-SoB), and Alzheimer’s Disease Cooperative Study—Activities Of Daily Living (ADCS-ADL) in the treatment group, compared with the placebo group [41–43].
- The incidence and severity of treatment-emergent adverse events or clinically important changes in safety assessments over 12 months.
- The changes in structural imaging measures, evaluated by entorhinal cortex and hippocampal volume, diffusion tensor imaging spectra, and magnetic resonance (MR) spectra from baseline to 12 months in the treatment group, compared with the placebo group.
- Establishing whether there is a reduction in microglial activation in subjects with mild AD following daily subcutaneous injection of liraglutide for 1 year, using translocator protein positron emission tomography (TSPO PET) scanning, compared with subjects receiving placebo injections, in a subgroup of participants.
- The change in the hippocampal, entorhinal, and other cortical regions’ tau deposition in the treatment group, compared to the placebo group, in a different subgroup of participants using tau PET.
- The changes in levels of cortical amyloid load in the treatment group, compared with the placebo group, in the same subgroup as the tau PET substudy.
- The change from baseline to 12 months in the composite score created using the support vector machine algorithm derived from cognitive tests, changes in MR imaging (MRI)-derived numerical summaries (hippocampal, temporal, and ventricular volume), changes in [18F]FDG-PET, Apolipoprotein E4 (ApoE4) status, and age.

The pharmacodynamics outcome is as follows:

- Plasma markers of neuroinflammation (proinflammatory and anti-inflammatory cytokines: IL-6, TNF-α, IL-8, IL-10, IL-12, CRP, TGF-β).

### Eligibility criteria

Inclusion and exclusion criteria are presented in Table 1.

**Table 1:** ELAD trial inclusion and exclusion criteria

Inclusion criteria	
1. Capable of giving and capacity to give informed consent	
2. An individual who can act as a reliable study partner with regular contact (a combination of face-to-face visits and telephone contact is acceptable) who has sufficient interaction with the participant to provide meaningful input into rating scales and, if necessary, supervise or perform the injections, as judged by the investigator	
3. Diagnosis of probable AD disease according to National Institute on Aging–Alzheimer’s Association (NIA-AA) criteria [50] or National Institute of Neurological and Communicative Disorders and Stroke–Alzheimer’s Disease and Related Disorders Association (NINCDS-ADRDA) criteria	
4. Age from 50 years	
5. Mini-Mental State Examination score of ≥ 15 and CDR-Global score of 0.5, 1, or 2 with capacity to consent, and the clinician anticipates that the participant will have capacity to complete the study	
6. Rosen Modified Hachinski Ischemic score ≤ 4	
7. On stable medication for 2 months before the screening visit; on or off cholinesterase inhibitors	
8. Fluency in English and evidence of adequate premorbid intellectual functioning	
9. Likely to be able to participate in all scheduled evaluations and complete all required tests	
Exclusion criteria	
1. Patients on treatment for diabetes mellitus	
2. Any contraindications to the use of liraglutide as per the summary of product characteristics (hepatic impairment, renal impairment with chronic kidney disease stage 4 and above (eGFR < 30 ml/min/1.73 m^2^), or inflammatory bowel disease). Patients with eGFR < 45 ml/min/1.73 m^2^ will have their renal function monitored very closely	
3. Significant neurological disease other than AD that may affect cognition	
4. MRI/CT showing unambiguous aetiological evidence of cerebrovascular disease with regard to their dementia or vascular dementia, fulfilling National Institute of Neurological Disorders and Stroke -Association Internationale pour la Recherche et l'Enseignement en Neurosciences (NINCDS-AIREN) criteria	
5. Current presence of a clinically significant major psychiatric disorder (e.g., major depressive disorder) according to the criteria of the *Diagnostic and Statistical Manual of Mental Disorders*, Fourth Edition (DSM-IV)	
6. Current clinically significant systemic illness that is likely to result in the patient’s condition deteriorating or affect the patient’s safety during the study	
7. History of epilepsy, where seizures or treatment could have contributed to cognitive impairment	
8. Treatment with immunosuppressive medications (e.g., systemic corticosteroids) within the last 90 days (topical and nasal corticosteroids and inhaled corticosteroids for asthma are permitted) or chemotherapeutic agents for malignancy within the last 3 years	
9. Myocardial infarction within the last 1 year	
10. History of cancer within the last 5 years, except localised skin cancer	
11. Other clinically significant abnormalities on physical, neurological, or laboratory examination that could compromise the study or be detrimental to the patient	
12. History of alcohol or drug dependence or abuse within the last 2 years	
13. Current use of anticonvulsant, anti-Parkinson’s disease medication	
14. Use of experimental medications for AD or any other investigational medication or device within 60 days. Participants who have been involved in a monoclonal antibody study are excluded unless it is known that they were receiving placebo in that trial	
15. Women of childbearing potential (women who could become pregnant will be required to use adequate contraception throughout the trial)	
16. Patients with a personal or family history of medullary thyroid carcinoma and patients with multiple endocrine neoplasia type 2	
17. Any contraindications to MRI scanning	

*AD* Alzheimer’s disease, *CDR* Clinical Dementia Rating, *CT* computed tomography, *eGFR* estimated glomerular filtration rate, *MRI* magnetic resonance imaging

### Study medication

Liraglutide, at doses up to 1.8 mg, has been approved in several countries, including the EU, Japan, Australia, and the USA, for the treatment of type 2 diabetes under the trade name Victoza®. In March 2015, the European Medicines Agency approved its use in obesity under the trade name Saxenda®, at doses up to 3 mg. The doses used in the ELAD trial are those approved for diabetes, as at the time of the study design liraglutide was only approved for clinical use in diabetes.

Pharmacokinetic data from the clinical development programme for liraglutide demonstrated that it is absorbed slowly (*t*_max_ = 8–12 h) and has a half-life of approximately 13 h. Liraglutide is thus suitable for once-daily subcutaneous injection given any time of the day, independent of meals [44].

Investigation of liraglutide metabolism in vitro and in healthy subjects has indicated that liraglutide is endogenously metabolised and that neither renal excretion nor hepatic extraction are major routes of clearance.

The pharmacokinetics of liraglutide has been investigated in human subjects with renal and hepatic impairment and has not raised any safety concerns. However, the therapeutic experience in subjects with hepatic or renal impairment is limited. The effects of age and gender on the pharmacokinetics of liraglutide have been investigated, and it was concluded that all participants, regardless of age or gender, should be dosed in accordance with the usual proposed dose regimen for liraglutide [45].

In the ELAD trial, participants will start with a dose of 0.6 mg liraglutide by subcutaneous injection into the abdomen, thigh, or upper arm. The dose will be escalated in weekly steps of 0.6 mg up to 1.8 mg once daily, if tolerated. Participants can stay on 1.2 mg if this is the maximum tolerated dose.

The study medication will be provided as prefilled pens so that the active treatment and placebo cannot be identified, guaranteeing that the study remains double-blinded. At the beginning of the study, the study drug will be administered subcutaneously under supervision. Participants and caregivers will be instructed in the administration and correct storage and handling of the pens.

Medication will be dispensed at weeks 0, 8, 16, 24, 32, 40, and 48 by each trial centre.

### Sample size

Landau et al. [46] used the [18F]FDG-PET imaging biomarker to monitor the progression of AD. At and after 12 months, they found a mean change in the [18F]FDG region of interest (ROI) of − 0.055, with an SD of 0.068. Assuming that the treatment reduces the mean change in the AD participants to − 0.025 (44% effect size), 82 participants are required per group to provide 80% power at a 5% significance level. Allowing for a drop-out of 15% over the study period, the trial requires 103 participants per group (206 in total).

## Trial conduct

### Study assessments

**Fig. 1:**
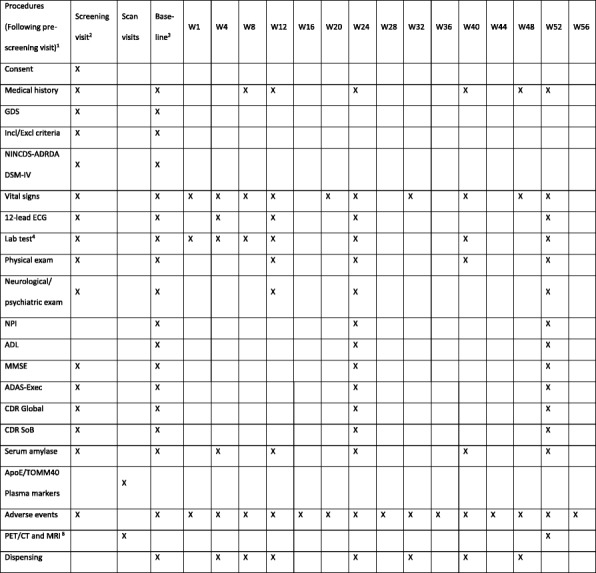
ELAD trial schedule of visits. ADAS-Exec Alzheimer’s Disease Assessment Scale—Cognitive Subscale and Executive domain scores of the Neuropsychological Test Battery, ApoE Apolipoprotein, CDR Clinical Dementia Rating, CT computed tomography, DSM-IV *Diagnostic and Statistical Manual of Mental Disorders*, Fourth Edition, ECG elecrocardiogram, Incl/Excl inclusion/exclusion, MMSE Mini Mental State Examination, MRI magnetic resonance imaging, NINCDS-ADRDA National Institute of Neurological and Communicative Disorders and Stroke–Alzheimer’s Disease and Related Disorders Association, PET positron emission tomography, SoB Sum of Boxes, W week

All participants will undergo the evaluations outlined in Fig. 1. Each visit during the double-blind phase will take place on the last day (± 4 days) of the study week. The week 16 (W16), week 28 (W28), week 36 (W36), and week 44(W44) visits will be a telephone call to check for any adverse events, with a follow-up visit at the study centre if needed. At the week 52 (W52) visit, final efficacy and safety assessments will be carried out and unused study medication will be collected. Four weeks after the end of the study, a final follow-up phone call will be made (W56). The clinical assessments will be done by a trained research nurse and a trained research doctor, when indicated. The neuropsychological assessments will be done by a trained rater at the research sites.

### Randomisation

Participants will be randomised to receive active drug or placebo with a 1:1 allocation ratio using stratified block randomisation with a fixed block size. The stratification factors are age and the Mini Mental State Examination. Randomisation will take place using an interactive voice response system. Mawdsleys [47] is the contractor responsible for randomising the ELAD participants and storing and distributing the drug. Figure 1 presents the schedule of enrolment for the trial.

### Withdrawals and unblinding

Participants who drop out of the ELAD study will be recorded as either defaulters or withdrawn. Defaulters are participants who withdraw their consent to participate in the trial, affecting both treatment and assessment. No further assessments can be made of these participants. Withdrawn are participants who are withdrawn from treatment at the discretion of the chief investigator because of clinical factors, poor compliance, or a change in circumstances. Withdrawn participants will remain in the study and trial assessments will still be undertaken.

The reason for dropping out will also be recorded. Investigators should be able to distinguish between drop-outs due to dementia-related factors (e.g., cognitive impairment), treatment-related factors (side effects), and incidental factors (concurrent physical illness or loss of study partners). Every effort will be made to collect outcome data on all participants who withdraw from treatment for whatever reason. If participants withdraw from treatment, all endpoint assessments should be carried out at the point of drop-out and at the 12-month endpoint. It may not be possible to complete some assessments with non-compliant participants. However, assessments will be carried out wherever possible and in all such cases at the 12-month endpoint.

As ELAD is a double-blind placebo-controlled trial, the participants, clinicians, statisticians, and chief investigator will be blinded to each patient’s treatment allocation. Unblinding will take place when the study has been completed and the data files have been verified. In a medical emergency, the designated person from the lead site at Imperial College, London will be able to break the randomisation code at the investigators’ request. A participant’s treatment assignment will only be unblinded when knowledge of the treatment is essential for their further medical management. Unblinding for any other reason will be considered a protocol violation.

### Data management

The ELAD data management plan is consistent with the MRC Guidelines for Good Clinical Practice in Clinical Trials (1998).

Clinical data will be entered into paper-based case report forms (CRFs), and then transferred into computers via Inform version 4.6. The database version is Oracle 10 g release 10.2.0.4.0 and is supplied by Oracle Corporation. The data manager will arrange appropriate quality assurance checks.

Participants eligible for study entry will be given a unique, sequential, centre-specific ELAD study identifier.

After each assessment, data will be entered into CRFs and the study database at each site. Immediately after each assessment, the data will be backed up electronically and securely stored locally. These files will be backed-up onto a password-protected environment on a weekly basis. Hard copies will be stored locally, compliant with the Data Protection Act (1998).

Every 2 weeks, data will be sent electronically to the data management centre at Imperial College London. Security will be maintained using email to and from password-protected, networked accounts and will comply with all regulatory requirements. The data management centre will merge data across centres and assessment points.

### Adverse events

Safety and tolerability assessments will consist of monitoring and recording all adverse events and serious adverse events, and the regular monitoring of vital signs. Clinically significant abnormalities in vital signs, laboratory evaluations, ECG recordings, and physical examinations will be recorded as adverse events and followed up as appropriate.

As far as possible, each adverse event will be described by its duration, severity grade, and relationship to the study drug, the action(s) taken, and, if relevant, the outcome.

Information about all serious adverse events will be collected on the ELAD trial. A serious adverse event is an undesirable sign, symptom, or medical condition that is fatal or life-threatening, requires hospitalisation, results in persistent or significant disability/incapacity, constitutes a congenital anomaly or birth defect, or is medically significant. A Suspected Unexpected Serious Adverse Reaction (SUSAR) is any adverse reaction that is classed as serious and suspected to be caused by the investigational medicine product that is not consistent with the information about it in the summary of product characteristics (i.e., it is suspected and unexpected). The trial protocol includes a list of known side effects for the drug in the study. If the event is not listed as expected or has occurred in a more serious form than anticipated, this will be considered a SUSAR. All serious adverse events will be followed up until the outcome of the event is “recovered”, “recovered with sequelae”, or “fatal”, and until all queries have been resolved. To ensure participants’ safety, each serious adverse event will be reported to the data monitoring committee within 24 h of the trial staff learning of its occurrence.

During each contact with trial site staff (site visits and telephone contacts), participants will be asked about adverse events and technical complaints. All adverse events, either observed by the site investigator or reported by the participant, will be reported by the investigator and evaluated by the Principal Investigator at each site.

### Data analysis

**Fig. 2:**
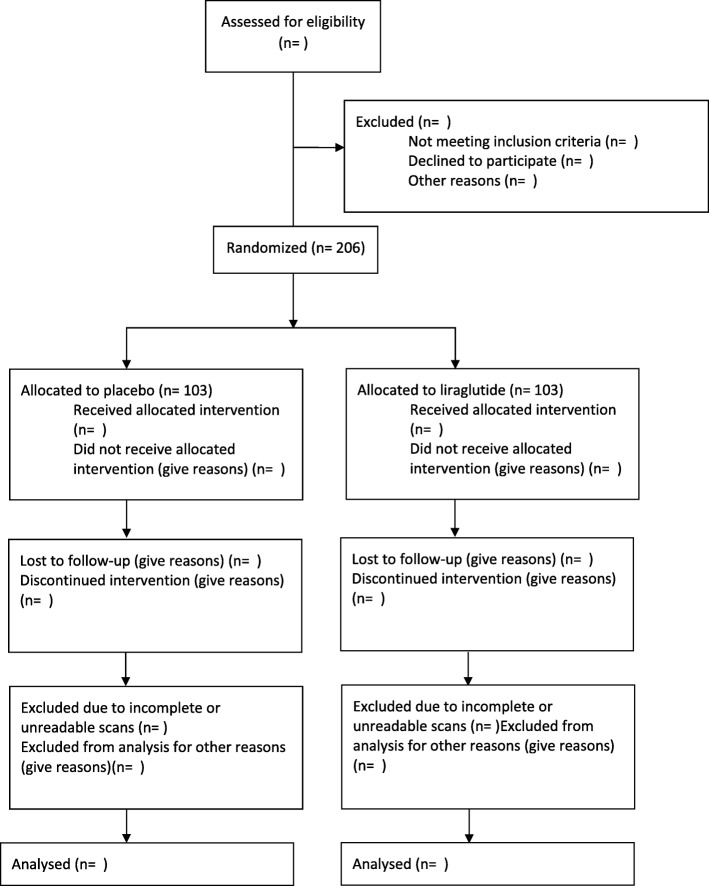
Schedule of enrolment

The primary analysis will be based on the modified intention-to-treat population. Participants will be analysed according to their allocated treatment group irrespective of what treatment they actually received. Patient throughput for the final analysis will be illustrated using a CONSORT [48] flow diagram (Fig. 2). Results will be presented as the adjusted mean difference in the change in cerebral glucose metabolic rate between randomised groups at 12 months, with 95% confidence intervals and associated two-sided *p* values. A full, detailed analysis plan will be prepared before the data are unblinded.

Patient demographic characteristics and other baseline information will be summarised by treatment group. Numbers (with percentages) for binary and categorical variables and mean (standard deviation) or median (interquartile or full range) for continuous variables will be presented. Differences in the change in FDG-ROI between randomised groups between baseline and 12 months will be assessed using analysis of covariance, adjusting for baseline values and stratification factors used in the randomisation process. The distribution of the change from baseline will be formally assessed for evidence of departure from normality. If necessary, data will be transformed or analysed using a non-parametric equivalent. The same approach will be used for the secondary outcomes measured at 12 months only. For outcomes measured on more than one occasion (e.g., ADAS-Exec), a mixed-effect model will be used. The data will be transformed or non-parametric methods used if the model assumptions are not met. Adverse events at 12 months will be analysed using Fisher’s exact test or the chi-squared test.

Every effort will be made to collect outcome data on all participants who withdraw from treatment for whatever reason. If participants withdraw from treatment, the endpoint assessments should all be carried out at the point of drop-out and at the 52-week endpoint. Although it may not be possible to complete some assessments of non-compliant participants, assessments should be carried out wherever possible and in all such cases at the 52-week endpoint.

The primary analysis will include all available data, including data from withdrawn participants irrespective of levels of treatment compliance. Assessing outcomes in participants with different levels of compliance will be performed as part of the per-protocol analysis with the per-protocol populations defined in the Statistical Analysis Plan. No primary analysis will be performed on defaulters since by definition they will not have completed the primary outcome (PET imaging) at 12 months (modified intention to treat analysis).

All reasons for withdrawing from the study will be summarised, overall and by arm.

A sensitivity analysis will be carried out on a per-protocol basis to examine the conclusions’ robustness to different assumptions about departures from the randomisation procedure. The per-protocol population is all participants who received the allocated treatment with no major protocol deviations and who have provided results at the end of follow-up assessment.

## Trial oversight

The chief investigator has overall responsibility for the conduct of the study. The trial management group has responsibility for the day-to-day management of the trial.

The trial steering committee, comprising independent clinicians and an independent statistician, acts as the oversight body for the trial on behalf of the sponsor. This committee will take responsibility for monitoring and guiding overall progress, scientific standards, and operational delivery and for protecting the rights and safety of the trial participants, throughout the trial.

An independent data monitoring committee (IDMC) will undertake ongoing reviews of the study’s safety. The committee composition, committee responsibilities, and a schedule for reviewing data have been approved and signed off by all committee members. The members include appropriately qualified clinicians and an independent statistician. The IDMC charter was prepared, reviewed, and approved ahead of the first committee meeting. The purpose of the charter is to describe the IDMC’s membership, terms of reference, roles, responsibilities, authority, decision-making, and relationships for this trial, including the timing of meetings, methods of providing information to and from the committee, frequency and format of meetings, statistical issues, and relationships with other committees. No formal interim analysis has been planned for ELAD, and one will only be conducted if the IDMC requests it (Additional file 1).

## Discussion

AD is a leading cause of disability worldwide. It is estimated that by 2050, 1 in 85 of the global population will be affected, resulting in a significant social and economic burden for healthcare systems. As the treatments currently available are only symptomatic and do not influence the course of the disease, the search for disease-modifying treatments is a priority. In the decade 2002–2012, more than 400 trials were performed in AD, testing 244 compounds. Of those, only memantine was approved for clinical use in AD, in 2003 [2]. The failure of the vast majority of anti-amyloid trials in AD suggests that other pathways should be explored in the search of an effective disease-modifying therapy.

There is convincing preclinical evidence that liraglutide has favourable effects on the neurodegenerative process of AD. Administering liraglutide in preclinical models reduces amyloid deposition and neuroinflammation, improves brain glucose metabolism and cognitive outcomes, and increases the proliferation of neuronal progenitor cells. Moreover, 6 months of liraglutide treatment in 20 AD participants prevented decline of brain glucose metabolism and other studies are investigating the potential for GLP-1 analogues in neurodegeneration. In this study, the participants are selected based on clinical diagnosis of AD, rather than on biomarker status, as suggested by more recent research guidelines [49]. As liraglutide works on multiple mechanisms, and not specifically on amyloid, including patients with a clinical diagnosis of AD will allow us to translate the findings and potential future treatment to the entire AD population diagnosed clinically rather than those with biomarker criteria.

Liraglutide is currently approved for the treatment of type 2 diabetes and obesity, where it shows a good safety profile and is well tolerated. If the ELAD trial is successful, liraglutide might represent an advance in the treatment of AD, to be further evaluated in larger studies, and might possibly have a positive impact on healthcare systems.

## Trial status

Recruitment is ongoing.

## Additional file

Additional file 1: SPIRIT 2013 checklist: items addressed in the clinical trial protocol. (DOC 121 kb)

## Abbreviations

AD: Alzheimer’s disease
ADAS: Alzheimer’s Disease Assessment Scale
ADCS-ADL: Alzheimer’s Disease Cooperative Study—Activities of Daily Living
ApoE: Apolipoprotein E4
CDR: Clinical Dementia Rating
CRP: C-reactive protein
FDG: Fluorodeoxyglucose
GLP-1: Glucagon-like peptide-1
IL: Interleukin
MRI: Magnetic resonance imaging
NIA-AA: National Institute on Aging–Alzheimer’s Association
NINCDS-ADRDA: National Institute of Neurological and Communicative Disorders and Stroke–Alzheimer’s Disease and Related Disorder Association
PET: Positron emission tomography
ROI: Region of interest
TGF-β: Transforming growth factor beta
TNF-α: Tumour necrosis factor alpha
TSPO: Translocator protein
