# Correction to: Evaluating the effects of the novel GLP-1 analogue liraglutide in Alzheimer’s disease: study protocol for a randomised controlled trial (ELAD study)

**DOI:** 10.1186/s13063-020-04608-4

**Published:** 2020-07-19

**Authors:** Grazia Daniela Femminella, Eleni Frangou, Sharon B. Love, Gail Busza, Clive Holmes, Craig Ritchie, Robert Lawrence, Brady McFarlane, George Tadros, Basil H. Ridha, Carol Bannister, Zuzana Walker, Hilary Archer, Elizabeth Coulthard, Ben R. Underwood, Aparna Prasanna, Paul Koranteng, Salman Karim, Kehinde Junaid, Bernadette McGuinness, Ramin Nilforooshan, Ajay Macharouthu, Andrew Donaldson, Simon Thacker, Gregor Russell, Naghma Malik, Vandana Mate, Lucy Knight, Sajeev Kshemendran, John Harrison, Christian Hölscher, David J. Brooks, Anthony Peter Passmore, Clive Ballard, Paul Edison

**Affiliations:** 1 Department of Medicine, Imperial College London, London, UK; 2 Centre for Statistics in Medicine, Nuffield Department of Orthopaedics, Rheumatology and Musculoskeletal Sciences, University of Oxford, Oxford, UK; 3 Southern Health NHS Foundation Trust, Havant, UK; 4 SW London and St George’s Mental Health Trust, London, UK; 5 Aston Medical school, Aston University, Birmingham, UK; 6 Brighton and Sussex University Hospitals NHS Trust, Brighton, UK; 7 South London and Maudsley NHS Foundation Trust, London, UK; 8 University College London and Essex Partnership University NHS Foundation Trust, Runwell, UK; 9 North Bristol NHS Trust, Bristol, UK; 10 Cambridgeshire and Peterborough NHS Foundation Trust, Peterborough, UK; 11 Black Country Partnership NHS Foundation Trust, West Bromwich, UK; 12 Northamptonshire Healthcare NHS Foundation Trust, Kettering, UK; 13 Lancashire Care NHS Foundation Trust, Preston, UK; 14 Nottinghamshire Healthcare NHS Foundation Trust, Nottingham, UK; 15 Centre for Public Health, Queen’s University Belfast, Belfast, UK; 16 Surrey and Borders Partnership NHS Foundation Trust, Leatherhead, UK; 17 NHS Ayrshire and Arran, Crosshouse, UK; 18 NHS Lanarkshire, Glasgow, UK; 19 Derbyshire Healthcare NHS Foundation Trust, Derby, UK; 20 Bradford District Care NHS Foundation Trust, Bradford, UK; 21 5 Boroughs Partnership NHS Foundation Trust, Warrington, UK; 22 Cornwall Partnership NHS Foundation Trust, Redruth, UK; 23 Somerset Partnership NHS Foundation Trust, Bridgwater, UK; 24 South Staffordshire and Shropshire Healthcare NHS Foundation Trust, Stafford, UK; 25 Alzheimer Center VUmc Amsterdam, Amsterdam, the Netherlands; 26 Institute of Psychiatry, Psychology & Neuroscience King’s College London, London, UK; 27 Research Department, Henan University of Chinese Medicine, Zhengzhou, China; 28 Newcastle University, Newcastle upon Tyne, UK; 29 School of Medicine, College of Biomedical and Life sciences, Cardiff University, Cardiff, CF14 4YS UK

**Correction to: Trials (2019) 20: 191**

**https://doi.org/10.1186/s13063-019-3259-x**

After publication of our article [1] the authors have notified us that the name of Prof. Christian Hölscher had been inadvertently forgotten when compiling the list of authors. Dr. Hölscher developed the concept of testing liraglutide in Alzheimer’s mouse model, has done the preclinical experiments, and is a co-applicant of the Alzheimer Society UK grant that partially funds the study.
